# Giant Splenorenal Shunt in a Young Patient with Autoimmune Hepatitis/Primary Biliary Cholangitis Overlap Syndrome and Portal Vein Thrombosis

**DOI:** 10.1155/2017/2167364

**Published:** 2017-02-20

**Authors:** F. Chegai, A. U. Cavallo, M. Forcina, V. Giuricin, F. Castellani, L. Greco, M. Manuelli, T. M. Manzia, G. Sergiacomi

**Affiliations:** ^1^Department of Diagnostic and Molecular Imaging, Radiation Therapy and Interventional Radiology, University Hospital Tor Vergata, Viale Oxford 81, 00133 Rome, Italy; ^2^Organ Transplantation Unit, University Hospital Tor Vergata, Rome, Italy

## Abstract

We present a case of giant Splenorenal Shunt (SRS) associated with portal vein thrombosis in a 37-year-old woman with a twelve-year history of autoimmune hepatitis/primary biliary cholangitis overlap syndrome. At the moment of the CT examination laboratory tests showed creatinine 1.5 mg/dl, bilirubin 1.5 mg/dl, INR 3, and Na 145 mmol/l and the Model End-Stage Liver Disease score was 24. Extensive calcified thrombosis causing complete occlusion of the portal vein lumen and partially occluding the origin of the superior mesenteric vein was present and a small calcified thrombus in the Splenic Vein lumen was also evident. SRS was located among the spleen hilum and the left kidney with a maximum diameter of 3.25 cm and was associated with dilatation of left renal vein and inferior vena cava. After a multidisciplinary evaluation the patient was put on the Regional Liver Transplant waiting list and liver transplantation was performed successfully. Although portal vein thrombosis and SRS are common occurrences in cirrhotic patients, the impact in the natural history of the disease is still unclear. Careful management and accurate imaging protocols are essential in the evaluation of those patients.

## 1. Introduction

Portal hypertension (PH) in patients with cirrhosis is caused by an increase in resistance to portal outflow and by a growth in splanchnic blood flow [1].

Likewise portal vein thrombosis (PVT) is frequent in these patients, particularly in the advanced stages of liver disease [2]. In a later phase of PH and cirrhosis specific changes occur, leading to a hyperkinetic circulation that raises cardiac output and reduces systemic vascular resistance as well as perfusion pressure [3]. The associated vasodilation and vasoconstriction with sinusoidal remodelling and related vascular distortion had a role in the pathophysiology of PH concurring to circulatory impairment and expansion of the collateral circulation. Frequent manifestations of PH include indeed the onset of large spontaneous shunts. As a consequence of increased vascular pressure, blood can be rerouted in the systemic circulation by collateral vessels which surround the portal vein, forming a “portal vein cavernoma” or by portosystemic shunts. Splenorenal Shunt (SRS) is defined as one of several spontaneous shunts, which may be created by reopening the embryonic venous route, in patients with portal hypertension. In patients with advanced liver disease who were candidates for liver transplantation (LT), the incidence varied from 3.8% to 60% [1]. This condition seems to be protective from variceal development by avoiding fatal bleeding but could complicate splenoliver surgery because of the difficulties of retroperitoneal dissection and preparation of the shunt. Few studies have evaluated the importance of SRS size changes after liver transplantation (LT). Recently Chikamori et al. [4] showed that the increase in the SRS/portal vein ratio is associated with reduction of liver function, hyperdynamic status, and narrowed arteriovenous oxygen content difference (*C*(a-v) O_2_).

Imaging of the portal venous system and large spontaneous shunts has a crucial role on evaluation of this clinical situation. Usually it is performed with color Doppler ultrasonography, contrast enhanced computed tomography (ceCT), and magnetic resonance imaging (MRI). Triphasic dynamic contrast enhanced CT is a useful tool for assessing abnormalities of the portal venous system.

We report the case of a young patient with autoimmune hepatitis/primary biliary cirrhosis overlap syndrome and PVT associated with solitary giant SRS.

## 2. Case Report

A 37-year-old woman with autoimmune hepatitis/primary biliary cholangitis overlap syndrome in good clinical conditions underwent routine CT examination.

The patient's clinical history dates back to 2004 when she noticed yellowish discoloration of sclera and skin associated with generalized itching, with no symptoms of biliary obstruction.

She did not report alcohol abuse and laboratory tests for HBV-HCV were negative. On physical examination, she was jaundiced and pale and abdominal examination revealed hepatomegaly.

Subsequent laboratory tests showed increased level of alkaline phosphatase (AP), Anti-Mitochondrial Antibodies (AMA) and Anti-Smooth Muscle Cells Antibodies (ASMA).

Histopathological examination after liver biopsy revealed mild to moderate chronic inflammatory portal tract infiltrates consisting of lymphocytes, polymorphs, and plasma cells and significant portal fibroplasia, bile ductular proliferation, and fibrosis.

After the clinical diagnosis the patient was treated with ursodeoxycholic acid (UDCA) and corticosteroids combination therapy with normalization of liver function tests in about six months.

In order to be placed in the waiting list for LT the patient underwent multislice CT using a 64-row MDCT scanner (Lightspeed VCT; General Electric, USA) with triphasic technique (30, 65, and 180 s) after intravenous administration of 120 ml of 350 mgI/ml iodine contrast media, injected at a rate of 3 ml/s with an automatic injector and followed by 20–30 ml of saline solution. CT was obtained with the following parameters: rotation time, 0.6 s; 2.5 to 5 mm thick sections with the possibility of back-reconstructions up to 0.6 mm; automatic milliamperage (mA) (min 300 mA, max 450 mA), and 120 kV. All reconstructed datasets were transferred to a dedicated offline workstation (VitreaWorkStation; Toshiba Medical, Japan) to obtain 2-dimensional Multiplanar Reconstruction (MPR) and Maximum Intensity Projection (MIP) and 3-dimensional (volume rendering, VR, technique) reconstructions.

At the moment of the CT examination blood laboratory tests showed the following: creatinine: 1.5 mg/dl (NV: 0.6–1.2 mg/dl); bilirubin: 1.5 mg/dL (NV: 3–1 mg/dl); INR: 3 (NV: 0.8–1.2); Na: 145 mmol/l (NV: 135–145 mmol/l).

**Figure 1:**
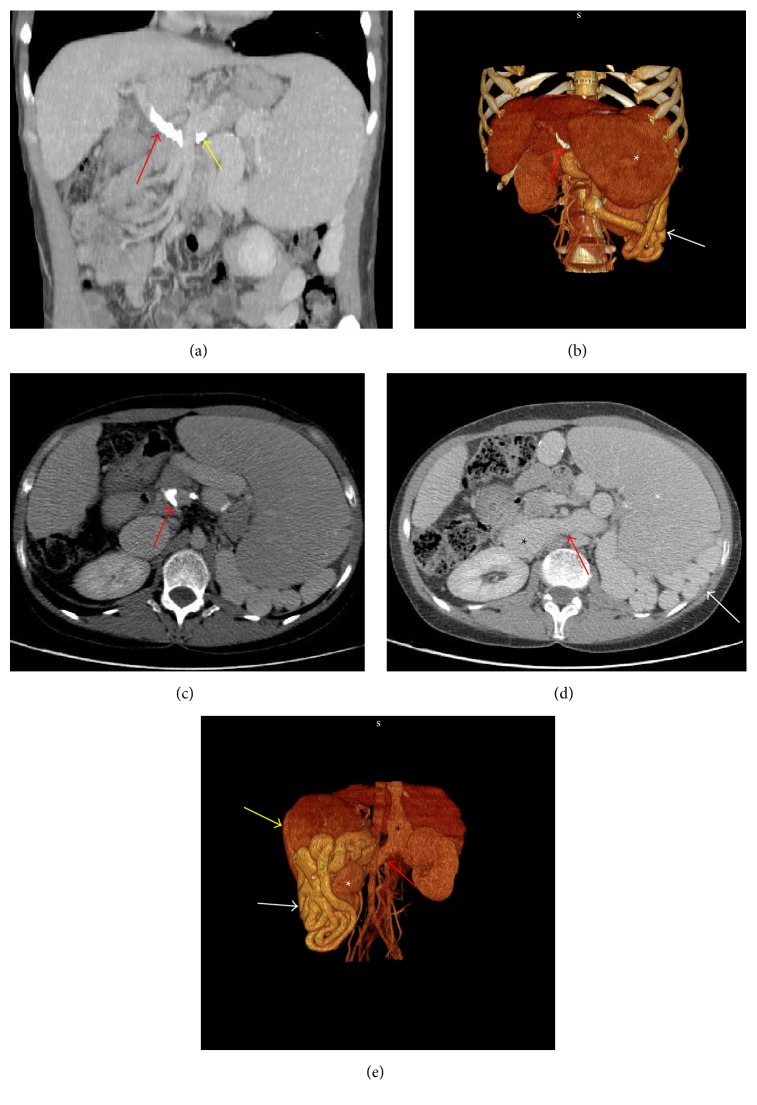
(a) MIP sagittal reconstruction showing portal vein calcified thrombosis. The thrombus appears as hyperdense structure occluding the portal vein lumen (red arrow). A little thrombus in the Splenic Vein is also evident (yellow arrow). (b) VR reformation showing portal vein calcified thrombosis (red arrow). The thrombus appears as hyperdense structure occluding the portal vein lumen (red arrow). Spleen (white star) and Splenorenal Shunt (white arrow) are also evident. (c) Axial view (portal phase) showing calcified thrombi partially occluding the superior mesenteric vein lumen (red arrow). (d) Axial view (portal phase) showing the Splenorenal Shunt (white arrow) behind the spleen (white star). Renal vein (red arrow) and Inferior Vena Cava (black star) dilatation is also evident. (e) VR reformation showing the Splenorenal Shunt (white arrow) among the spleen (yellow arrow) and the left kidney (white star). Renal vein (red arrow) and Inferior Vena Cava (black star) dilatation is also evident.

CT showed liver with reduced size and hypertrophy of caudate lobe and no evidence of intrahepatic focal lesions. Extensive calcified thrombosis causing complete occlusion of the portal vein lumen and partially occluding the proximal superior mesenteric vein (SMV) (Yerdel grade 3) [2] was present (Figures 1(a)–1(c)) and a small calcified thrombus in the Splenic Vein (SV) lumen was also evident (Figure 1(a)). Among the spleen hilum and the left kidney a massive SRS was evident: hypodense structure in basal scans, made of tortuous tubular structures detectable in the venous phase scans (Figures 1(d) and 1(e)). Multiplanar Reconstruction allowed accurately measuring the maximum axial diameter. The shunt had a maximum diameter of 3.25 cm and was associated with dilatation of left renal vein and Inferior Vena Cava (Figures 1(d) and 1(e)).

A subsequent color Doppler evaluation showed a forward flow direction in the SV.

A multidisciplinary meeting including a radiologist, a hepatologist, and a transplant surgeon evaluated risks and benefits of LT. Because of the young age and the 20% estimated survival expectancy without transplantation according to Mayo Clinic Risk score, the patient was put on the Regional Liver Transplant waiting list.

At the time of LT, the Model End-Stage Liver Disease (MELD) score was 24 and the Mayo Risk Score was 9.0. Surgeons performed the transplant procedure using a 1992-Belghiti piggyback technique. Due to the presence of calcific portal vein sclerosis extending into the proximal SMV a portal thrombectomy was excluded. Hence, after side-to-side vena cava anastomosis, SRS was sectioned at the confluence to the left renal vein and the renal side was brought behind the stomach, in order to perform a T-T anastomosis between portal vein and venous shunt.

The procedure was performed successfully and then the patient was kept in the Intensive Care Unit (ICU) 24 hours. Postoperative course was uneventful and the patient was discharged on postoperative day 7 with excellent graft function and in good clinical conditions.

## 3. Discussion

The portal venous system comprises the veins draining the abdominal tract of the digestive system, including the lower esophagus, except the lower anal canal. The main tributaries of the portal vein are the splenic, left gastric, right gastric, superior mesenteric, paraumbilical, and cystic veins [3]. In patients with portal hypertension blood flow is diverted in the systemic vessels by different kinds of collateral pathways, including esophageal, paraesophageal, coronary gastric, inferior phrenic, paraumbilical, abdominal wall, splenorenal, gastrorenal, retrocaval, and mesocaval collateral.

Angiography was considered the gold standard for the evaluation of varices but, with the availability of cross-sectional imaging techniques, the demonstration of collateral portal vessels can be obtained in all parts of the abdomen and thorax without the risks associated with the catheterization. Although endoscopy is considered the best modality for detection of esophageal varices, angiography and computed tomography (CT) can demonstrate more reliably varices in other locations [5]; MR imaging can ease the diagnosis and the evaluation of these conditions and in particular cases is complementary to CT evaluation [6]. With CT imaging techniques, varices appear as smooth, well-defined structures, round, tubular, or serpentine in shape, with homogeneous attenuation, often surrounded by fat in the retroperitoneum, greater and lesser omentum, and mesentery, with a similar enhancement to the adjacent vessels after the administration of contrast medium, which is useful for distinguishing them by bowel loops, tumor masses, or adenopathy [5].

Color Doppler ultrasound is the first-line diagnostic approach for the detection of PVT; the sensitivity and specificity vary from 66% to 100%, depending on the expertise of the individual examiner and the extent of PVT, and are reduced when bowel gas and obesity are present. The main limitation is delimiting the extension of the thrombi to SV or SMV [7]. When technical conditions are adverse contrast enhanced computed tomography (CT) and magnetic resonance imaging (MRI) are both good options to delineate the extent and the precise location of the thrombus. Unenhanced CT can display fresh thrombi as hyperdense, but this condition does not often occur and contrast medium administration is often required [8]. CT and US examinations are also useful for detecting calcifications within the wall of the vessel or the thrombus, which can eventually complicate the LT procedure [8]. The impact of the development of PVT on the natural history of liver cirrhosis is unclear. Different studies did not show a real impact of PVT on the progression of the disease [9, 10]. Even if PVT does not seem to increase the wait list mortality in patient scheduled for LT [11, 12], it represents a relevant occurrence in patients undergoing LT because it is associated with more difficult surgery, more postoperative complications, higher in-hospital mortality rates [2], and reduced 5-year survival rates [13].

In this particular case PVT was associated with SRS, a quite common consequence of portal hypertension.

Due to the long course of the disease and the progressive increase of portal resistance it is likely that the development of a calcified thrombus in the portal vein can be considered as a slow and progressive event that has advanced up to determine the complete occlusion of the venous trunk.

The presence of SRS and hemodynamic changes related to it might explain the slight symptoms of the patient, as well as the modest alterations of laboratory tests.

In patients with advanced cirrhosis, in fact, the incidence of SRS is approximately 35% [13]. Portal venous flow and velocity are reduced in patients with cirrhosis and large SRS, compared with normal subjects and patients with chronic hepatitis [14] due to the venous blood shunting in the systemic circulation which determines an increase of cardiac output. The enhanced portal blood flow tends to counterpoise the hypotensive effect of the portosystemic shunt. As portal blood flow grows, collateral blood flow multiplies and is nearly totally shunted in the systemic circulation [15].

Due to these hemodynamic changes SRS prevents the formation of gastroesophageal varices but does not reduce the risk of bleeding [16, 17] and is associated with an increased risk of complications such as hepatic encephalopathy [18] and HCC [15].

Chikamori et al. [4] claimed that SRS/portal vein ratio can be an indicator of hyperdynamic status in patients with liver cirrhosis. Authors demonstrated that there is a relationship between the SRS/PV ratio and the degree of hyperdynamic circulatory status in patients with cirrhosis.

SRS management in LT remains controversial, as there is still no consensus on indications to LT and modalities of care. In our case ceCT with MPR and volume rendering reconstruction helped surgeons to properly plan the LT.

In our case ceCT with MPR and volume rendering reconstruction helped surgeons to properly plan the LT

## 4. Conclusions

Careful management is necessary for patients with PVT and SRS because of the influence on hemodynamic parameters, mainly cardiac output, and correlation with increased risk of complication such as hepatic encephalopathy.

Imaging plays a pivotal role in the evaluation and follow-up of cirrhotic patients, and ceCT is especially useful in the evaluation of vascular alterations such as PVT and SRS.
